# The Antibacterial Activity of Honey Derived from Australian Flora

**DOI:** 10.1371/journal.pone.0018229

**Published:** 2011-03-28

**Authors:** Julie Irish, Shona Blair, Dee A. Carter

**Affiliations:** School of Molecular Bioscience, University of Sydney, Camperdown, Australia; National Institutes of Health, United States of America

## Abstract

Chronic wound infections and antibiotic resistance are driving interest in antimicrobial treatments that have generally been considered complementary, including antimicrobially active honey. Australia has unique native flora and produces honey with a wide range of different physicochemical properties. In this study we surveyed 477 honey samples, derived from native and exotic plants from various regions of Australia, for their antibacterial activity using an established screening protocol. A level of activity considered potentially therapeutically useful was found in 274 (57%) of the honey samples, with exceptional activity seen in samples derived from marri (*Corymbia calophylla*), jarrah (*Eucalyptus marginata*) and jellybush (*Leptospermum polygalifolium*). In most cases the antibacterial activity was attributable to hydrogen peroxide produced by the bee-derived enzyme glucose oxidase. Non-hydrogen peroxide activity was detected in 80 (16.8%) samples, and was most consistently seen in honey produced from *Leptospermum* spp. Testing over time found the hydrogen peroxide-dependent activity in honey decreased, in some cases by 100%, and this activity was more stable at 4°C than at 25°C. In contrast, the non-hydrogen peroxide activity of *Leptospermum* honey samples increased, and this was greatest in samples stored at 25°C. The stability of non-peroxide activity from other honeys was more variable, suggesting this activity may have a different cause. We conclude that many Australian honeys have clinical potential, and that further studies into the composition and stability of their active constituents are warranted.

## Introduction

The use of honey as a wound dressing is gaining acceptance in modern medicine as a result of its antimicrobial activity and wound healing properties. In particular, certain types of honey exhibit broad-spectrum antimicrobial activity and are effective against antibiotic resistant bacterial pathogens [1], [2], [3], [4], [5]. Honey-based wound care products have been registered with medical regulatory authorities as wound care agents in Australia, Canada, the European Union, Hong Kong, New Zealand and the USA. In most instances these products use manuka honey from New Zealand or the equivalent honey produced from other *Leptospermum* species in Australia.

Honey has several properties that contribute to its antimicrobial activity. In most honeys, low pH and high osmolarity are combined with the enzymatic production of hydrogen peroxide that exerts an antimicrobial effect [6], [7]. Phytochemical components derived from the floral source of the honey can confer additional activity that is stable in the presence of catalase, an enzyme that destroys hydrogen peroxide [8]. This non-peroxide activity was first identified in manuka (*Leptospermum scoparium*) honey from New Zealand where it is often marketed as the Unique Manuka Factor (UMF®).

Variations in the type and level of antimicrobial activity in honey are associated with their floral source. However, while some floral sources appear to be associated with particular levels of hydrogen peroxide activity, variation in this activity among honeys from within the same floral species has also been observed [9], [10], [11]. This may be due to the geographical location of the floral source and the prevailing environmental conditions, which affect the physiology of the floral species [12], or to bee-related factors such as age or colony health, which may affect the production or activity of glucose oxidase (the enzyme responsible for hydrogen peroxide production in honey) [13], [14], [15], [16]. The precise mechanisms determining the level of this type of activity are yet to be elucidated.

Honeys with non-peroxide antimicrobial activity are more closely associated with floral source, being generally derived from *Leptospermum* species [8], [9], although this type of activity has also been found in a small number of non-*Leptospermum* honeys [9], [17], [18], [19]. In a clinical setting where honey is used as a topical antimicrobial and wound dressing, non-peroxide activity may be advantageous as it is not destroyed by catalase present in body fluids, and is unaffected by gamma irradiation [20], allowing these honeys to be sterilized for medicinal use. The compound primarily responsible for non-peroxide activity in New Zealand manuka honey has recently been identified as methylglyoxal (MG) [21], [22], which is derived from dihydroxyacetone, a compound present in high levels in manuka nectar [23]. The reasons for varying dihydroxyacetone levels in different plants are not yet understood.

An agar well diffusion method to determine the antibacterial activity of honey with reference to phenol [9] has become the *de facto* standard in medical honey testing, and is used commercially to assign a UMF® value to medicinal honeys. This method is a simple and rapid way to screen large numbers of honey samples for antibacterial activity; however, it does not discriminate between individual antibacterial factors and their relative contributions to overall antibacterial activity. Using this method, Allen *et al*. [9] conducted a survey of 345 New Zealand honeys and found wide variation in hydrogen peroxide-dependent antibacterial activity, both within and among floral sources. Non-peroxide activity was identified in a significant proportion of samples of manuka (*L. scoparium*) and Viper's bugloss (*Echium vulgare*) honeys. A survey of 179 non-manuka New Zealand honeys by Brady *et al*. [10] also found wide variation in hydrogen peroxide-dependent activity, and non-peroxide activity was not detected in any samples. The only study using the phenol equivalence method conducted outside New Zealand is a small survey of 30 Portuguese honeys from several floral sources [17]. This study revealed low levels of hydrogen peroxide-dependent activity in all samples, and low levels of non-peroxide activity in six samples, primarily from *Lavandula* species.

Australia is home to diverse and unique floral resources that are exploited by the beekeeping industry. No published data exist on the antimicrobial activity of most Australian honey, and the benefits of this knowledge to both the apiary industry and the health care sector are clear. Therefore, the aim of this study was to survey a wide range of Australian honey sourced from different native and exotic flora for antimicrobial activity. Honey samples were tested for their levels of total antibacterial activity and non-peroxide activity, and correlations were investigated between the type and level of antimicrobial activity and the floral source of the honey, its region of origin and the age of the honey sample. Over half of the honey samples tested had antibacterial activity in the range considered to be therapeutically useful. Exceptional hydrogen peroxide-dependent antibacterial activity was found in honey derived from *Eucalyptus marginata* (jarrah) and *Corymbia calophylla* (marri) from Western Australia, and very highly active non-peroxide honeys were produced from *Leptospermum* species, particularly *L. polygalifolium,* growing in the coastal New South Wales-Queensland border region. Although floral source and region were clearly important in the production of active honey, the level of activity varied widely among samples and changed during storage.

## Materials and Methods

### Honey samples

**Figure 1:**
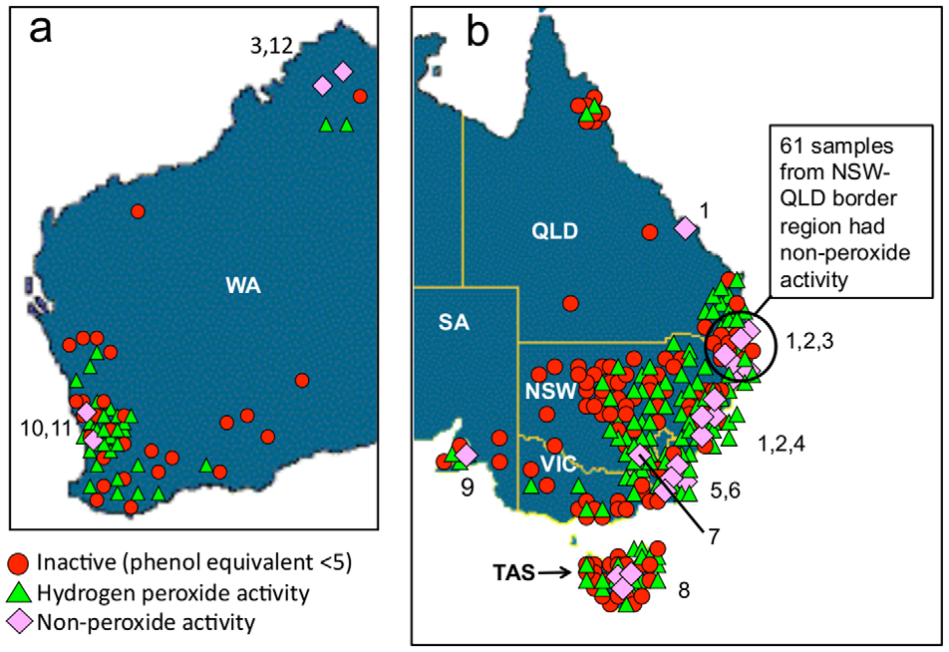
Location and activity of honey samples. a) Samples from west Australia (WA = Western Australia); b) Samples from east Australia and Tasmania (QLD = Queensland; NSW = New South Wales; SA = South Australia; VIC = Victoria; TAS = Tasmania). Numbers indicate floral source of the honey samples with non-hydrogen peroxide activity: 1. *Leptospermum* spp. alone; 2. *Leptospermum* spp. in mixed flora; 3. Unspecified flora; 4. *Melaleuca* and brush box; 5. Spotted gum; 6. Forest red gum; 7. Clover; 8. Wild flowers; 9. Messmate stringybark; 10. Orchard; 11. Coastal Moort; 12. *Melaleuca* alone.

A total of 477 honey samples were received from beekeepers and honey companies throughout Australia between March 2005 and June 2007. A map indicating the location of the honey samples is shown in Figure 1. Each sample was assigned a unique reference number and details provided by the beekeepers were entered into a database (see Table S1). Honey samples were stored in glass or plastic containers at room temperature in the dark. Comvita Wound Care 18+ honey (Comvita New Zealand Ltd., Paengaroa, New Zealand), a pure manuka honey from New Zealand with non-peroxide antibacterial activity equivalent to at least 18% phenol was used as a positive control. This honey is commercially available as a wound dressing and is registered with appropriate regulatory bodies in Australia, New Zealand, the USA and the EU.

Identification of the floral source of the honey was performed by the beekeepers based on the availability of flora for nectar foraging, location of the apiary and organoleptic characteristics of the honey. Where beekeepers supplied only the common name of the floral source, the scientific name was determined from the Australian Plant Common Name Database [24], Australian Plant Name Index [25] and/or floral distribution maps [26], [27], [28], [29], [30], where possible.

### Phenol equivalence assay for antibacterial activity

Antibacterial activity of honey samples with reference to phenol was determined as described by Allen *et al.* [9]. An 18 h culture of *Staphylococcus aureus* ATCC 9144 (Oxoid, Hampshire, UK) grown in tryptone soya broth (TSB; Oxoid) was adjusted to an absorbance of 0.5 at 540 nm. Large assay plates (245×245 mm; Corning Inc., Corning, NY, USA) were prepared with 150 ml of nutrient agar (Becton, Dickinson and Company, Sparks, MD, USA) that had been seeded with 100 µl of the prepared *S. aureus* culture. Plates were stored inverted at 4°C for use the next day, when 64 wells were cut into the agar with a sterile 8 mm diameter cork borer, over a 25 mm grid. Each well was numbered, in duplicate, using a quasi-Latin square that enabled the duplicate samples to be placed randomly on the plate.

Honey samples were prepared freshly for each assay by adding 10 ml of sterile deionised water to 10 g of well-mixed honey. One ml of each honey solution was mixed with 1 ml of sterile deionised water for total activity testing, or 1 ml of a freshly prepared 5600 U/ml catalase solution (Sigma, St Louis, MO, USA) for non-peroxide activity testing. A 100 µl aliquot of each solution was placed in wells of the assay plate, in duplicate.

Phenol (BDH, VWR International Ltd., Poole, UK) standards of 2%, 3%, 4%, 5%, 6%, and 7% were freshly prepared every four weeks in sterile deionised water and stored at 4°C. Aliquots of 100 µl of each solution were placed in duplicate wells of the assay plate. Negative controls of sterile deionised water and catalase solution were included in duplicate wells of each assay plate. Comvita Wound Care 18+ honey was prepared as for other honey samples for use as a positive control. The plates were incubated at 37°C for 18 h.

The diameter of each zone of inhibition was measured in two directions at right angles to each other using Vernier callipers. The mean diameter of the zone of inhibition around each well was calculated and squared, and a standard curve was generated of phenol concentration against the mean squared diameter of the zone of inhibition. The activity of each diluted honey sample was calculated using the standard curve. To account for the dilution and density of honey, this figure was multiplied by 4.69 (based on a mean honey density of 1.35 g/ml, as determined by [31]), and the activity of the honey was then expressed as the equivalent phenol concentration (% w/v) [9], [31]. Each honey sample was tested on at least two separate occasions, and the mean phenol equivalence was used in further analysis.

### The effect of sample age on antibacterial activity

A subset of 20 honey samples (10 with hydrogen peroxide activity only and 10 with non-peroxide activity) were selected for retesting following storage of aliquots in the dark at 4°C and at 25°C for 8 to 22 months after the first test. Honey samples were re-tested in duplicate on two separate occasions, and the mean phenol equivalence was used in further analysis.

### Data analysis

The data consisted of four categorical variables (floral source, floral origin (native, exotic, or mixed), region, sample age), and two main response variables (total activity and non-peroxide activity). All data were analysed qualitatively, with the exception of the change in antibacterial activity over time. Statistical analysis of change in activity over time was performed with Minitab 14 statistical software (Minitab Inc. Pennsylvania, USA), using the Wilcoxon signed ranks test. To aid statistical analysis, honeys with antibacterial activity below the limit of detection of the assay (approximately 5% phenol equivalent) were assigned a value of 5, although these values are reported as <5 where appropriate.

## Results

### Reproducibility of the phenol equivalence assay

Comvita Wound Care 18+ honey was used as a positive control to monitor the reproducibility of the phenol equivalence assay. This commercially available product is standardised such that its non-peroxide activity is at least 18% (w/v) phenol equivalent. Over the course of this study, the mean total activity of this honey was 17.9±0.9% phenol equivalent, and the mean non-peroxide activity was 17.3±1% phenol equivalent. Day to day variation in activity was within ±2% phenol equivalent of the specified 18%. This range was exceeded on only one occasion and all honey samples in that plate were retested. Replicate tests of individual honey samples were also within the range of ±2% phenol equivalent.

### Total antibacterial activity of honey samples

Antibacterial activity equivalent to at least 10% (w/v) phenol should provide therapeutic benefits as an antimicrobial [32]. The antibacterial activity of the 477 honey samples was therefore divided into categories of undetectable activity (<5% phenol equivalent), low activity (5–10% phenol equivalent), potentially therapeutically beneficial activity (10–20% phenol equivalent) and high activity (>20% phenol equivalent).

**Figure 2:**
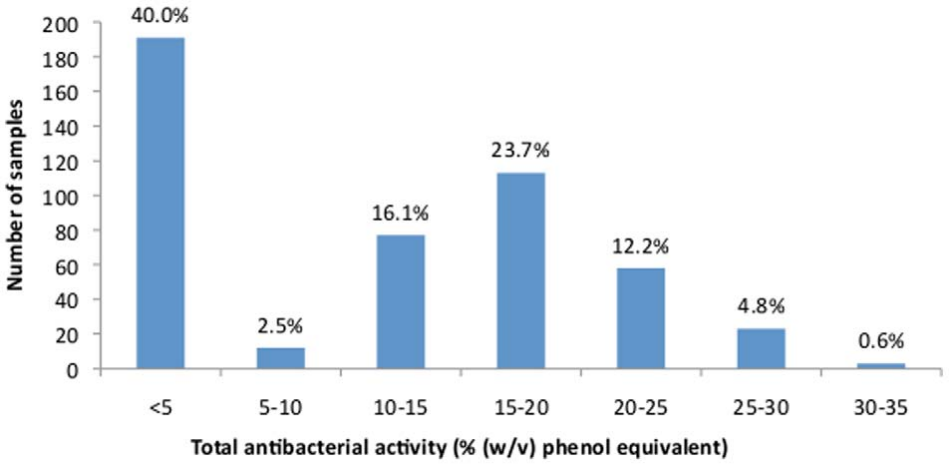
Total antibacterial activity of Australian honey samples. Graph shows combined peroxide and non-peroxide dependent activity in 477 honey samples collected from Australian floral sources, divided into increments of (w/v) phenol equivalent.

The total antibacterial activity (encompassing both hydrogen peroxide-dependent and non-peroxide activity) of the 477 honey samples is shown in Figure 2. The average total activity was 10.6±9.5% phenol equivalent (range: <5–34.3; median: 13). Detectable antibacterial activity was found in 286 (60%) of the honey samples, with an average total activity of 17.8±5% phenol equivalent (range: 7.4–34.3; median: 17.1). A total of 274 (57%) of the honey samples had activity of ≥10% phenol equivalent and could be considered to be therapeutically useful.

The 477 honey samples were derived from 142 different floral sources, including combinations of known flora, as well as unspecified mixed flora. The majority of honey samples (372 samples = 78%) were derived from native Australian flora; 80 samples (16.8%) were of mixed origin and were likely to contain native floral species; and 25 samples (5.2%) were derived from exotic floral species. Table 1 shows the median antibacterial activity of honeys from floral sources with three or more samples, ranked by median total activity (for activity of all samples see Table S1).

**Table 1:** Total antibacterial activity of honey samples from floral sources with a sample size ≥3, ranked by median activity.

Floral source: Common name (Scientific name)	No. samples	No. (%) with detectable activity^1^	Total activity^1^	
			Range	Median
Marri (*Corymbia calophylla*)	8	7 (88)	<5–29.7	25.7
Jarrah (*Eucalyptus marginata)*	19	18 (95)	<5–31.4	25.1
Jelly bush and heath flora (*Leptospermum polygalifolium* and unknown species)	3	3 (100)	17.3–19.9	19.8
Spotted gum (*Corymbia maculata)*	4	4 (100)	14.7–25.1	18.9
Tea tree and paperbark (*Leptospermum semibaccatum* and *Melaleuca nodosa*)	4	4 (100)	18.1–19.6	18.8
Jelly bush (*L. polygalifolium*)	29	28 (97)	<5–26.2	17.9
Jelly bush, tea tree (*Leptospermum* sp.)	14	12 (86)	<5–25.8	17.8
Mixed flora, Sydney metropolitan region	32	25 (78)	<5–29.8	15.9
Lemon-scented tea tree (*Leptospermum liversidgei*)	5	5 (100)	14.0–24.5	15.7
Red stringybark (*Eucalyptus macrorhyncha)*	9	5 (56)	<5–26.1	15.3
Crow's ash and jelly bush (*Guioa semiglauca* and *L. polygalifolium*)	3	2 (67)	<5–19.4	15.2
Banksia (*Banksia* sp.)	25	22 (88)	<5–24.1	15.0
Jelly bush mix (*L. polygalifolium* and *Leptospermum speciosum*	3	3 (100)	14.2–14.7	14.6
Clover (*Trifolium repens*)	3	2 (67)	<5–16.3	14.3
Manuka (*Leptospermum scoparium*)	11	9 (82)	<5–16.3	13.1
Paperbark, tea tree (*Melaleuca* sp.)	22	18 (82)	<5–19.6	12.8
Mugga ironbark (*Eucalyptus sideroxylon*)	3	3 (100)	9.7–12.3	11.7
Mixed wildflowers, Tasmania	5	4 (80)	<5–16.1	11.6
Feather bush (*Micromyrtus ciliata*)	3	2 (67)	<5–13.6	11.5
Other mixed or unknown flora	35	19 (54)	<5–24.6	9.9
Messmate stringybark (*Eucalyptus obliqua*)	5	3 (60)	<5–15.2	9.8
Snow gum (*Eucalyptus pauciflora*)	3	2 (67)	<5–10.5	8.7
Tea tree and paperbark (*Leptospermum laevigatum* and *Melaleuca nodosa*)	4	2 (50)	<5–16.3	7.7
Tea tree, paperbark (*Melaleuca quinquenervia*)	3	2 (67)	<5–21.9	7.4
Paterson's curse, Salvation Jane (*Echium plantagineum*)	4	2 (50)	<5–15.6	6.3
Leatherwood (*Eucryphia lucida*)	11	4 (36)	<5–17.5	<5
Wandoo (*Eucalyptus wandoo*)	7	2 (29)	<5–18.7	<5
Lemon-scented tea tree and pink bloodwood (*Leptospermum liversidgei* and *Corymbia intermedia*)	17	3 (18)	<5–14.6	<5
Eucalyptus (*Eucalyptus sp.*)	15	5 (33)	<5–24.9	<5
Parrot bush (*Dryandra sessilis*)	3	1 (33)	<5–21.0	<5
Coastal tea tree (*Leptospermum laevigatum*)	4	1 (25)	<5–21.4	<5
Mixed rainforest flora, Queensland	3	1 (33)	<5–16.2	<5
Blue gum (*Eucalyptus globulus*)	3	1 (33)	<5–15.3	<5
Yellow box (*Eucalyptus melliodora*)	4	1 (25)	<5–12.7	<5
Saw banksia (*Banksia serrata*)	4	0 (0)	<5	<5
Coriander (*Coriandrum sativum*)	3	0 (0)	<5	<5
Heather bush (*Thryptomene micrantha*)	3	0 (0)	<5	<5
Tea tree and yellow box (*Leptospermum* sp. and *E. melliodora)*	3	0 (0)	<5	<5
Macadamia (*Macadamia integrifolia*)	3	0 (0)	<5	<5
Red mallee (*Eucalyptus oleosa*)	4	0 (0)	<5	<5
Powderbark (*Eucalyptus accedens*)	3	0 (0)	<5	<5

1. Activity calculated as % (w/v) phenol equivalent

### Honey with non-peroxide antibacterial activity

Non-peroxide activity was detected in 80 honey samples (16.8%), with a mean of 15.6±4.7% phenol equivalent (range: 8.1–25.9; median: 15.4). A summary of these honeys is shown in Table 2, and a map indicating their floral source and region of origin is shown in Figure 1. Samples that were derived from *Leptospermum* floral species or contained *Leptospermum* as part of a mixed floral source comprised 77.5% of honey samples with detectable non-peroxide activity (mean non-peroxide activity of *Leptospermum*-containing honeys: 17.2±4.1% phenol equivalent; range: 9.8–25.9; median: 16.4). Eighteen (22.5%) of the honey samples derived from flora other than *Leptospermum* also exhibited non-peroxide activity (average non-peroxide activity of non-*Leptospermum* honeys: 10.1±1.7% phenol equivalent; range: 8.1–15.9; median: 10). Non-peroxide activity in *Leptospermum*-containing honeys generally comprised a higher proportion of the total antibacterial activity (up to 100%) than in non-*Leptospermum* honeys.

**Table 2:** Honey samples exhibiting non-peroxide antibacterial activity.

Floral source	No. samples tested	No. (%) samples with non-peroxide activity	Mean non-peroxide activity ± SD* (mean % of total activity ± SD)
*Leptospermum* spp. alone	68	48 (71)	17.9±4.2 (94.9±6.4)
*Leptospermum* spp. in mixed flora	44	14 (32)	14.7±2.6 (85.8±11.8)
Tasmanian wildflowers	5	3 (60)	12.7±2.7 (97.2±2.6)
Forest red gum	2	1 (50)	11.2±1.1 (46.5±7.7)
*Melaleuca* and brush box	2	1 (50)	10.5±0.7 (51.8±7.2)
Spotted gum	4	3 (75)	10.1±0.3 (51.1±14.3)
*Melaleuca* alone	26	1 (4)	9.7±0.9 (66.8±2.2)
Unspecified flora	72	5 (7)	9.2±0.9 (78.4±18.3)
Clover	3	1 (33)	9.2±0.1 (64.0±3.7)
Orchard	2	1 (50)	9.1±0.2 (28.4±1.4)
Messmate stringybark	6	1 (17)	9.0±0.4 (59.2±0)
Coastal moort	1	1 (100)	8.8±0.3 (67.4±11.6)

*Calculated as % (w/v) phenol equivalent for samples within a floral source with non-peroxide activity.

The non-peroxide antibacterial activity of honey derived from single *Leptospermum* species is shown in Table 3. Non-peroxide activity was evident in honey from *L. polygalifolium*, *L. liversidgei*, *L. laevigatum* and some unspecified species. These honeys were collected primarily in the Northern Rivers region of New South Wales and the adjacent Southeast Coast region of Queensland, with one sample from the Capricornia region of Queensland (Figure 1). *Leptospermum* honeys collected from other states and regions did not exhibit non-peroxide activity.

**Table 3:** Non-peroxide antibacterial activity and region of origin of honey derived from single *Leptospermum* species.

*Leptospermum* species	Region*	No. samples tested	No. (%) samples with non-peroxide activity	Mean non-peroxide activity ± SD
*L. polygalifolium*	Northern Rivers NSW	28	27 (96)	18.9±3.9
	Capricornia QLD	1	1 (100)	21.1
*L. liversidgei*	Northern Rivers NSW	5	5 (100)	16.1±4.4
*L. laevigatum*	Northern Rivers NSW	1	1 (100)	19.7
	Central VIC	2	0 (0)	<5
	Hunter NSW	1	0 (0)	<5
*L. scoparium*	Southeast Huon, Channel and Lower Derwent Valley TAS	1	0 (0)	<5
	Northeast and Flinders Island TAS	10	0 (0)	<5
*L. flavescens*	Illawarra NSW	1	0 (0)	<5
*L. continentale*	Central VIC	2	0 (0)	<5
Unspecified *Leptospermum sp.*	Northern Rivers NSW	6	5 (83)	16.2±5.1
	Southeast Coast QLD	4	4 (100)	19.5±5.4
	Illawarra NSW	1	0 (0)	<5
	Metropolitan NSW	1	0 (0)	<5
	Northern Tablelands NSW	1	0 (0)	<5
	Murraylands SA	1	0 (0)	<5

*NSW: New South Wales; QLD: Queensland; SA: South Australia; TAS: Tasmania; VIC: Victoria; see Figure 1 for map locations.

### The effect of sample age and storage temperature on antibacterial activity

The majority of honey samples were collected from hives between 2001 and 2007, and tested between 2006 and 2007. No collection date was specified for 66 samples, and one sample was collected in 1978. Scatter plots of antibacterial activity vs. sample age for all honeys of known age showed no correlation between antibacterial activity and age of the honey sample (r^2^ = 0.0062 for total antibacterial activity; r^2^ = 0.0072 for non-peroxide activity).

Aliquots of a subset of 20 honey samples (10 samples with hydrogen peroxide-dependent activity only and 10 samples with non-peroxide activity) were stored in the dark at 25°C and 4°C, and were re-tested between 8 and 22 months after first testing (Table 4; see Table S2 for the full dataset). Repeat testing found the median total antibacterial activity of honeys exhibiting only hydrogen peroxide-dependent activity significantly decreased over time at both 25°C and 4°C (Wilcoxon signed ranks test *P*<0.01). This loss of activity was significantly greater after storage at 25°C compared to storage at 4°C (Wilcoxon signed ranks test *P*<0.01). All honeys exhibiting only hydrogen peroxide-dependent activity decreased in activity, with an average of loss of 9.5% phenol equivalent, and two samples lost all detectable activity after storage at 25°C. For the 10 samples exhibiting non-peroxide activity, the median total and non-peroxide activity did not change significantly over time at either storage temperature (Wilcoxon signed ranks test *P*>0.05). However, among these it appeared that the three honey samples derived from pure *L. polygalifolium* all increased in activity, particularly those stored at 25°C (+16 to +34% change in total activity and +13 to +37% change in non-peroxide activity), while the five samples that were from sources excluding *L. polygalifolium* showed only very minor increases or decreased in activity during storage (–4 to –34% change in total activity and +2 to –16% change in non-peroxide activity at 25°C).

**Table 4:** Change in antibacterial activity of honey samples following storage.

Floral source: Common name (Scientific name) [age at 1^st^ assay in months]	Activity pre-storage^1^		Months in storage	% Change in activity post-storage at 25°C		% Change in activity post-storage at 4°C	
	Total	Non-peroxide		Total	Non-peroxide	Total	Non-peroxide
Red stringybark (*Eucalyptus macrorhyncha*) [10]	26.1	<5	17	-34	0	-20	0
Mixed urban flora [11]	17.0	<5	16	-28	0	-22	0
Viper's bugloss and lucerne (*Echium vulgare* and *Medicago sativa*) [22]	17.2	<5	17	-41	0	-28	0
Grey ironbark (*Eucalyptus paniculata*) [1]	15.6	<5	16	-100	0	-14	0
Forest red gum (*Eucalyptus tereticornis*) [5]	18.3	<5	16	-26	0	-27	0
Turpentine (*Syncarpia glomulifera*) [42]	24.7	<5	22	-100	0	-43	0
Bloodwood (*Corymbia gummifera*) [3]	23.3	<5	16	-45	0	-10	0
Avocado (*Persea americana*) [3]	21.8	<5	16	-42	0	-22	0
Mixed urban flora [45]	24.6	<5	23	-33	0	-5	0
Red stringybark (*Eucalyptus macrorhyncha*) [42]	24.6	<5	22	-48	0	-42	0
Jelly bush (*Leptospermum polygalifolium*) [<1]	15.9	15.3	18	+35	+37	+7	+10
Jelly bush (*L. polygalifolium*) [46]	17.2	17.1	17	+29	+28	+8	+5
Jelly bush (*L. polygalifolium*) [54]	23.4	23.4	9	+16	+13	+9	+9
Jelly bush and crow's ash (*L. polygalifolium* and *Guioa semiglauca*) [46]	19.4	13.3	21	-12	+23	-24	+3
Jelly bush and tea tree (*L. polygalifolium* and *Leptospermum whitei*) [9]	13.9	13.2	20	-12	-12	0	-4
Clover (*Trifolium repens*) [9]	14.3	9.2	23	-34	+2	-37	-3
Mixed flora [3]	9.9	8.5	21	-4	+1	-13	-2
Paperbark and brush box (*Melaleuca* sp. and *Lophostemon confertus*) [7]	20.8	10.5	21	-15	-11	-4	-6
Lemon-scented tea tree (*Leptospermum liversidgei)* [8]	14.6	13.4	21	-21	-16	-5	-7
Lemon-scented tea tree (*L. liversidgei*) [17]	24.5	23.6	11	-9	-12	+1	+1

Determined as (w/v) phenol equivalent.

## Discussion

The integration of honey into modern medicine as a therapeutic agent requires that medicinal honey products exhibit a high level of antimicrobial activity that is consistent and standardised, as with any other medicinal product. It is therefore of critical importance to the apicultural, horticultural and medical industries to identify floral species that give rise to honey with consistently high activity. This study is the first to provide a broad overview of the antibacterial activity of Australian honey from a wide variety of floral sources. Results show that these honeys exhibit a wide range of antibacterial activity, and the majority have potential for therapeutic use.

### Honey derived from certain Australian flora possesses exceptional antibacterial activity

Honey with non-peroxide activity is highly sought after in the medicinal honey market due to its potential clinical advantages. This study demonstrates that the prevalence of non-peroxide activity among Australian honey samples, and the level of this activity, exceeds that reported in honey from other countries. Non-peroxide activity was identified in 70.6% of Australian *Leptospermum* honey samples tested, with a median of 16.7% phenol equivalent (Table 2).

The methylglyoxal (MG) content of Australian *Leptospermum* honeys has not yet been investigated, and it is possible that this compound is present in similar or higher levels than in manuka honey. Non-peroxide activity was strongly associated with *Leptospermum* honeys collected from the Northern Rivers region of New South Wales and the adjacent Southeast Coast region of Queensland (Figure 1), indicating that these regions are a potentially valuable source of therapeutically beneficial honey. Among the *Leptospermum* species, *L. polygalifolium* (jellybush) produced honey that was particularly high in activity (Table 3). Although *L. scoparium* (manuka) is the primary source of honey with non-peroxide activity in New Zealand, none of the 11 samples of *L. scoparium* honey from Australia had detectable non-peroxide activity. These findings suggest that environmental conditions in different regions play a role in the relationship between floral source and non-peroxide antibacterial activity, or alternatively that different regions contain as yet uncharacterised subspecies of *Leptospermum* that are responsible for providing honeys with non-peroxide activity. In New Zealand, different concentrations of phenolic compounds, including MG, are found in *L. scoparium* honeys collected from different regions, with the potential to affect antibacterial activity [33]. Further botanical and genetic studies of Australian *Leptospermum* species are required to elucidate these differences, and may inform studies aimed at cultivating particular plant species in productive regions for highly active medicinal honey.

Exceptionally high activity was also seen in hydrogen peroxide-dependent honeys derived from marri (*C. calophylla*; median activity 25.7, maximum 29.7) and jarrah (*E. marginata*; median activity 25.1, maximum 31.4) from Western Australia. To our knowledge these are the most potent antibacterial honeys yet reported. Very high activity was also seen in 22% of honeys from the Sydney metropolitan region, indicating that highly active honey may be obtained from a number of different environments. Although there is a focus in the literature on the antimicrobial activity of *Leptospermum* honey, many *in vitro* studies investigating the antimicrobial activity of honey have found that manuka honey and honey with similar levels of hydrogen peroxide activity are equally effective against bacterial pathogens [2], [3], [4], [5], [34], [35], [36]. Honeys with hydrogen peroxide-dependent activity are more effective than manuka honey at inhibiting dermatophyte fungi [37] and species of the yeast *Candida* [38], indicating that these honeys may be more broad spectrum and valuable as antifungal agents than manuka honey.

### Antibacterial activity is highly variable

The antibacterial activity of the Australian honey samples tested exhibited a distinctly bimodal distribution (Figure 2), with peaks at 0–5% and 15–20% (w/v) phenol equivalent. This suggests that the antibacterial activity in fresh honey is largely “all-or-nothing”, although what governs this is not known as there was substantial variation in activity both among and within floral sources. Of the 41 floral sources represented by three or more honey samples, only 6 produced uniformly active honey, and none of the honeys with more than 10 samples were consistently active (Table 1). At the other end of the scale, few of the multiply sampled floral sources produced uniformly inactive honey (Table 1). Plant-derived factors that contribute to the antimicrobial activity in honey may be influenced by local environmental conditions such as climate, water and nutrient availability [12], and entomological factors may also contribute to activity [39]. The complex interplay of plant species, plant physiology, growth conditions, seasonal variations and bee physiology make it difficult to predict whether or not a given honey sample is likely to have antimicrobial activity.

A remarkable finding of the current study was that even honeys produced in one location at one time could vary in activity. In one example, 22 *Banksia* honey samples obtained following a single flowering event were tested, with each honey sample collected from a separate hive in the same apiary (samples B11–B32; Table S1). Total antibacterial activity among 21 of these samples ranged from 11.4 to 19.2% phenol equivalent, and one sample had no detectable activity. Similarly, 18 *Melaleuca* honey samples that had been collected from separate hives in a single apiary included four inactive samples, with the remainder ranging in total activity from 10.8 to 14.3% phenol equivalent (samples T11–T28; Table S1). This suggests that entomological differences can have a substantial role in the activity of honey, even more so than the floral source. The health of individual bee colonies and the age of foraging workers may affect foraging activity or the secretion of enzymes responsible for antibacterial activity, including glucose oxidase [13], [14], [15], [16]. In addition, since truly monofloral honeys are often practically impossible to obtain, different foraging preferences among colonies may result in honey produced from the nectar of numerous floral species [40], thereby altering the overall activity. Floral sources of honey are primarily identified as the dominant species in flower at the time, and mixed floral sources may have been more prevalent than was reported by beekeepers. This is of particular interest for non-*Leptospermum* honeys exhibiting non-peroxide activity, as there is the possibility that they contain some nectar from *Leptospermum* species. This was considered unlikely in the current study, however, since most were from regions where *Leptospermum* is either not present or would not be in flower when the bees were foraging. It is also possible that *Leptospermum* honey with non-peroxide activity that was collected in the Northern Rivers or Southeast Coast regions may contain nectar from *L. polygalifolium*, even if beekeepers identified the dominant floral source as a different *Leptospermum* species. A more detailed investigation of the floral sources of these honeys, perhaps using pollen analysis, is warranted.

Non-peroxide activity was identified in 18 honey samples not derived from *Leptospermum* flora (Table 2; Figure 1), including the majority of honeys derived from spotted gum and Tasmanian wildflowers (3/4 and 3/5 of the honeys sampled, respectively). On the whole, however, this activity was sporadic, with no clear link to a particular floral source or geographic region. Tests on the stability of honey following storage found the samples with non-peroxide activity derived from clover, mixed flora and paperbark/brush box, as well as samples from *L. liversidgei,* either remained relatively stable or declined in activity over time, while the three honey samples derived from only *L. polygalifolium* increased in activity (Table 4). Many beekeepers find that non-peroxide activity increases over time [41], which may correspond to an increase in Maillard reaction products including MG [23], [33], [42]. The fact that this did not happen in non-peroxide honeys that were derived from plants other than *L. polygalifolium* suggests that at least some of the activity in these honeys is due to antimicrobial compounds other than MG. Bee defensin-1 and other peptides, along with various phenolics, have been found in different honey samples and have been proposed to convey antimicrobial effects [19], [39], [43], [44]. Whether any of these occur in the Australian non-peroxide honeys remains to be determined.

The stability of the antibacterial activity of honey over time has implications for the shelf life of medicinal honey products. In the case of hydrogen peroxide-dependent honeys this is likely to be due to the instability of glucose oxidase, the enzyme responsible for hydrogen peroxide production, which is influenced by various factors including pH and exposure to light [45]. Enzyme stability is often affected by temperature, and the loss in activity was mitigated to some extent by storage at 4°C (Table 4). Extra care in the handling and storage of honeys with hydrogen peroxide-dependent activity may therefore be necessary if these are to be used in the clinical setting. Regardless of the reason behind any change in activity, honeys that are used in laboratory tests over prolonged periods should be tested regularly to ensure that the level of activity has remained constant. Degradation of activity over time does not preclude the use of honey as an antimicrobial agent, since all medicinal products have a shelf life and many require refrigeration. However, a greater understanding of the time frame and the storage conditions that affect loss of activity are vital in producing a standardised medicinal product.

### Conclusions

This study has provided a broad overview of the antibacterial activity of Australian honey and shown that many honeys have potential for therapeutic use as antibacterial agents. Jarrah and marri honeys have exceptional levels of hydrogen peroxide-dependent activity, and non-peroxide activity in Australian *Leptospermum* honeys is comparable to that found in New Zealand manuka honey. These findings indicate that there is an opportunity for Australian apiarists to share in the lucrative medicinal honey market. However, the factors affecting antibacterial activity in honey are complex, numerous, and not solely dependent on the floral source. This prevents generic statements being made regarding the activity of honey derived from a given floral source, and indicates the need to test individual batches of honey for their level of antibacterial activity before they are designated as therapeutic products.

## Supporting Information

Supporting Table S1 Complete list of honey samples included in survey including floral source, geographic region and antibacterial activity (total and non-peroxide) for each sample.
(XLS)

Supporting Table S2 Change in antibacterial activity of honey samples following storage at 25°C and 4°C (complete data set).
(DOCX)

## References

[1] Blair SE, Cokcetin NN, Harry EJ, Carter DA (2009). The unusual antibacterial activity of medical-grade
*Leptospermum* honey: antibacterial spectrum, resistance
and transcriptome analysis.. Eur J Clin Microbiol Infect Dis.

[2] Cooper RA, Wigley P, Burton NF (2000). Susceptibility of multiresistant strains of *Burkholderia
cepacia* to honey.. Lett Appl Microbiol.

[3] Cooper RA, Halas E, Molan PC (2002). The efficacy of honey in inhibiting strains of
*Pseudomonas aeruginosa* from infected
burns.. J Burn Care Rehabil.

[4] Cooper RA, Molan PC, Harding KG (2002). The sensitivity to honey of Gram-positive cocci of clinical
significance isolated from wounds.. J Appl Microbiol.

[5] French VM, Cooper RA, Molan PC (2005). The antibacterial activity of honey against coagulase-negative
staphylococci.. J Antimicrob Chemother.

[6] Bang LM, Buntting C, Molan P (2003). The effect of dilution on the rate of hydrogen peroxide
production in honey and its implications for wound healing.. J Altern Complement Med.

[7] White JW, Subers MH, Schepartz AI (1963). The identification of inhibine, the antibacterial factor in
honey, as hydrogen peroxide and its origin in a honey glucose-oxidase
system.. Biochim Biophys Acta.

[8] Molan PC, Russell KM (1988). Non-peroxide antibacterial activity in some New Zealand
honeys.. J Apic Res.

[9] Allen KL, Molan PC, Reid GM (1991). A survey of the antibacterial activity of some New Zealand
honeys.. J Pharm Pharmacol.

[10] Brady N, Molan P, Bang L (2004). A survey of non-manuka New Zealand honeys for antibacterial and
antifungal activities.. J Apic Res.

[11] Molan PC, Smith IM, Reid GM (1988). A comparison of the antibacterial activity of some New Zealand
honeys.. J Apic Res.

[12] Price JN, Morgan JW (2006). Variability of plant fitness influences range expansion of
*Leptospermum scoparium*.. Ecography.

[13] Ohashi K, Natori S, Kubo T (1999). Expression of amylase and glucose oxidase in the hypopharyngeal
gland with an age-dependent role change of the worker honeybee (*Apis
mellifera* L.).. Eu J Biochem.

[14] Janmaat AF, Winston ML, Ydenberg RC (2000). Condition-dependent response to changes in pollen stores by honey
bee (*Apis mellifera*) colonies with different parasitic
loads.. Behav Ecol Sociobiol.

[15] Mattila HR, Otis GW (2006). Effects of pollen availability and Nosema infection during the
spring on division of labor and survival of worker honey bees (Hymenoptera:
Apidae).. Environ Entomol.

[16] Yang X, Cox-Foster DL (2005). Impact of an ectoparasite on the immunity and pathology of an
invertebrate: evidence for host immunosuppression and viral
amplification.. Proc Natl Acad Science USA.

[17] Henriques A, Burton NF, Cooper RA (2005). Antibacterial activity of selected Portuguese
honeys.. J Apic Res.

[18] Cabrera L, Cespedes E, Nava R, de Rodriguez GO (2006). Non-peroxide antibacterial activity in Zulia
honeys.. Revista Cientifica-Facultad De Ciencias Veterinarias.

[19] Mundo M, Padilla-Zakour O, Worobo R (2004). Growth inhibition of foodborne pathogens and food spoilage
organisms by select raw honeys.. Int J Food Microbiol.

[20] Molan PC, Allen KL (1996). The effect of gamma-irradiation on the antibacterial activity of
honey.. J Pharm Pharmacol.

[21] Adams CJ, Boult CH, Deadman BJ, Farr JM, Grainger MNC (2008). Isolation by HPLC and characterisation of the bioactive fraction
of New Zealand manuka (*Leptospermum scoparium*)
honey.. Carbohydrate Res.

[22] Mavric E, Wittmann S, Barth G, Henle T (2008). Identification and quantification of methylglyoxal as the
dominant antibacterial constituent of Manuka (*Leptospermum
scoparium*) honeys from New Zealand.. Mol Nutr Food Res.

[23] Adams CJ, Manley-Harris M, Molan PC (2009). The origin of methylglyoxal in New Zealand manuka
(*Leptospermum scoparium*) honey.. Carbohydrate Res.

[24] Australian National Botanic Gardens (2008). Australian Plant Common Name Database.. http://www.anbg.gov.au/common.names.

[25] Centre for Plant Biodiversity Research (2008). Australian Plant Name Index, IBIS database.. http://www.cpbr.gov.au/cgi-bin/apni.

[26] Brooker MIH, Kleinig DA (2001). Field Guide to Eucalypts, Vol. 2 - South-western and Southern
Australia..

[27] Brooker MIH, Kleinig DA (2004). Field Guide to Eucalypts, Vol. 3 - Northern
Australia..

[28] Brooker MIH, Kleinig DA (2006). Field Guide to Eucalypts, Vol. 1 - South-eastern
Australia..

[29] Clemson A (1985). Honey and pollen flora..

[30] Centre for Plant Biodiversity Research (2008). Australia's Virtual Herbarium (map output).. http://www.cpbr.gov.au/cgi-bin/avh.cgi.

[31] Allen KL, Molan PC, Reid GM (1991). The variability of the antibacterial activity of
honey.. Apiacta.

[32] Cooper RA, Jenkins L (2009). A comparison between medical grade honey and table honeys in
relation to antimicrobial efficacy.. http://www.woundsresearchcom/content/a-comparison-be-tween-medicalgrade-honey-and-table-honeys-relation-antimicrobial-efficacy.

[33] Stephens JM, Schlothauer RC, Morris BD, Yang D, Fearnley L (2010). Phenolic compounds and methylglyoxal in some New Zealand manuka
and kanuka honeys.. Food Chem.

[34] Allen KL, Molan PC (1997). The sensitivity of mastitis-causing bacteria to the antibacterial
activity of honey.. N Z J Agric Res.

[35] Cooper RA, Molan PC (1999). The use of honey as an antiseptic in managing
*Pseudomonas* infection.. J Wound Care.

[36] Cooper RA, Molan PC, Harding KG (1999). Antibacterial activity of honey against strains of
*Staphylococcus aureus* from infected
wounds.. J R Soc Med.

[37] Brady NF, Molan PC, Harfoot CG (1996). The sensitivity of dermatophytes to the antimicrobial activity of
manuka honey and other honey.. Pharm Sci.

[38] Irish J, Carter D, Shokohi T, Blair S (2006). Honey has an antifungal effect against *Candida*
species.. Med Mycol.

[39] Kwakman PHS, te Velde AA, de Boer L, Speijer D, Vandenbroucke-Grauls CMJE (2010). How honey kills bacteria.. FASEB.

[40] Dag A, Fetscher AE, Afik O, Yeselson Y, Schaffer A (2003). Honey bee (*Apis mellifera*) strains differ in
avocado (*Persea americana*) nectar foraging
preference.. Apidologie.

[41] Somerville DC (2008). A study of New Zealand beekeeping: lessons for
Australia.. Rural Industries Research and Development Corporation Publication No
08/060.

[42] Castro-Vazquez L, Diaz-Maroto MC, Gonzalez-Vinas MA, de la Fuente E, Perez-Coello MS (2008). Influence of storage conditions on chemical composition and
sensory properties of citrus honey.. J Agric Food Chem.

[43] Gallardo-Chacon JJ, Casellies M, Izquierdo-Pulido M, Rius N (2008). Inhibitory activity of monofloral and multifloral honeys against
bacterial pathogens.. J Apicul Res.

[44] Molan PC (1992). The antibacterial activity of honey. 1. The nature of the
antibacterial activity.. Bee World.

[45] White JW, Subers MH (1964). Studies on honey Inhibine. 4. Destruction of the peroxide
accumulation system by light.. J Food Sci.

[46] Flores MJ, Ehrlich SD, Michel B (2002). Primosome assembly requirement for replication restart in the
*Escherichia coli* holDG10 replication
mutant.. Molecular Microbiology.

