# Supplementary material for: The Antibacterial Activity of Honey Derived from Australian Flora
Source: PLoS One. 2011 Mar 28;6(3):e18229. doi: 10.1371/journal.pone.0018229 (PMC3065476; doi:10.1371/journal.pone.0018229)
Supplement: Supporting Table S2 — Change in antibacterial activity of honey samples following storage at 25°C and 4°C (complete data set). (DOCX) [file pone.0018229.s002.docx]

**Table S2: Change in antibacterial activity of honey samples following storage**

| Floral source | Pre-storage | | | Post-storage (room temperature) | | | Post-storage (4°C) | |
| --- | --- | --- | --- | --- | --- | --- | --- | --- |
| Common name (Scientific name) [Sample designation]* | Sample age (months) | Total activity | Non-peroxide activity | Sample age (months) | Total activity (% change in activity) | Non-peroxide activity (% change in activity) | Total activity (% change in activity) | Non-peroxide activity (% change in activity) |
| Red stringybark (*Eucalyptus macrorhyncha*) [SB14] | 10 | 26.1 | <5 | 27 | 17.3 (-34) | <5 (0) | 20.9 (-20) | <5 (0) |
| Mixed urban flora [P34] | 11 | 17.0 | <5 | 27 | 12.2 (-28) | <5 (0) | 13.2 (-22) | <5 (0) |
| Viper’s bugloss and lucerne (*Echium vulgare* and *Medicago sativa*) [BL2] | 22 | 17.2 | <5 | 39 | 10.2 (-41) | <5 (0) | 12.4 (-28) | <5 (0) |
| Grey ironbark (*Eucalyptus paniculata*) [IB6] | 1 | 15.6 | <5 | 17 | <5 (-100) | <5 (0) | 13.4 (-14) | <5 (0) |
| Forest red gum (*Eucalyptus tereticornis*) [BG4] | 5 | 18.3 | <5 | 21 | 13.5 (-26) | <5 (0) | 13.3 (-27) | <5 (0) |
| Turpentine (*Syncarpia glomulifera*) [T10] | 42 | 24.7 | <5 | 64 | <5 (-100) | <5 (0) | 14.1 (-43) | <5 (0) |
| Bloodwood (*Corymbia gummifera*) [BW3] | 3 | 23.3 | <5 | 19 | 12.8 (-45) | <5 (0) | 21.0 (-10) | <5 (0) |
| Avocado (*Persea americana*) [A1] | 3 | 21.8 | <5 | 19 | 12.6 (-42) | <5 (0) | 17.0 (-22) | <5 (0) |
| Mixed urban flora [P33] | 45 | 24.6 | <5 | 67 | 16.4 (-33) | <5 (0) | 23.3 (-5) | <5 (0) |
| Red stringybark (*Eucalyptus macrorhyncha*) [SB8] | 42 | 24.6 | <5 | 64 | 12.9 (-48) | <5 (0) | 14.4 (-42) | <5 (0) |
| Jelly bush (*L. polygalifolium*) [JB21] | <1 | 15.9 | 15.3 | 19 | 21.5 (+35) | 20.9 (+37) | 17.0 (+7) | 16.8 (+10) |
| Jelly bush (*L. polygalifolium*) [JB15] | 2 | 17.2 | 17.1 | 19 | 22.2 (+29) | 21.9 (+28) | 18.5 (+8) | 17.9 (+5) |
| Jelly bush (*L. polygalifolium*) [JB28] | 54 | 23.4 | 23.4 | 63 | 27.2 (+16) | 26.5 (+13) | 25.6 (+9) | 25.5 (+9) |
| Jelly bush and crow’s ash (*L. polygalifolium* and *Guioa semiglauca*) [CL1] | 2 | 19.4 | 13.3 | 23 | 17.0 (-12) | 16.3 (+23) | 14.7 (-24) | 13.7 (+3) |
| Jelly bush and tea tree (*L. polygalifolium* and *Leptospermum whitei*) [L17] | 9 | 13.9 | 13.2 | 29 | 12.3 (-12) | 11.6 (-12) | 13.9 (0) | 12.7 (-4) |
| Clover (*Trifolium repens*) [C12] | 9 | 14.3 | 9.2 | 32 | 9.4 (-34) | 9.4 (+2) | 9.0 (-37) | 8.9 (-3) |
| Mixed flora [K4] | 3 | 9.9 | 8.5 | 24 | 9.5 (-4) | 8.6 (+1) | 8.6 (-13) | 8.3 (-2) |
| Paperbark and brush box (*Melaleuca* sp. and *Lophostemon confertus*) [MB1] | 7 | 20.8 | 10.5 | 28 | 17.7 (-15) | 9.4 (-11) | 20.0 (-4) | 9.9 (-6) |
| Lemon-scented tea tree (*Leptospermum liversidgei)* [L13] | 8 | 14.6 | 13.4 | 29 | 11.6 (-21) | 11.3 (-16) | 13.9 (-5) | 12.5 (-7) |
| Lemon-scented tea tree (*Leptospermum liversidgei*) [L11] | 17 | 24.5 | 23.6 | 28 | 22.2 (-9) | 20.7 (-12) | 24.8 (+1) | 23.7 (+1) |

Based on designations given in Supporting Table S1
